# Real-world response assessment of immune checkpoint inhibition: comparing iRECIST and RECIST 1.1 in melanoma and non-small cell lung cancer patients

**DOI:** 10.1007/s00330-024-11060-4

**Published:** 2024-09-18

**Authors:** Christian Nelles, Moritz Gräf, Pascale Bernard, Thorsten Persigehl, Nils Große Hokamp, David Zopfs, David Maintz, Nicole Kreuzberg, Jürgen Wolf, Paul J. Bröckelmann, Simon Lennartz

**Affiliations:** 1 Institute for Diagnostic and Interventional Radiology, Faculty of Medicine and University Hospital Cologne, University of Cologne, Cologne, Germany; 2 Department I of Internal Medicine, Center for Integrated Oncology Aachen Bonn Cologne Düsseldorf (CIO ABCD), Faculty of Medicine and University Hospital Cologne, University of Cologne, Cologne, Germany; 3 Department of Dermatology and Venereology, Skin Cancer Center, Center for Integrated Oncology Aachen Bonn Cologne Düsseldorf (CIO ABCD), Faculty of Medicine and University Hospital Cologne, University of Cologne, Cologne, Germany

**Keywords:** Response evaluation criteria in solid tumors, Immune checkpoint inhibitors, Melanoma, Carcinoma (non-small-cell lung)

## Abstract

**Objectives:**

To compare immune response evaluation criteria in solid tumors (iRECIST) and response evaluation criteria in solid tumors (RECIST) 1.1 for response assessment of immune checkpoint inhibitor (ICI) therapy in a real-world setting in patients with melanoma and non-small cell lung cancer (NSCLC).

**Methods:**

Two-hundred fifty-two patients with melanoma and NSCLC who received CTLA-4 inhibitor ipilimumab or PD-1 inhibitors nivolumab or pembrolizumab and who underwent staging CT of the chest and abdomen were retrospectively included. Treatment response evaluation according to the RECIST 1.1 and iRECIST guidelines was performed for all patients. Response patterns, as well as overall response rate (ORR), disease control rate (DCR), and time to progression (TTP), were compared between RECIST 1.1 and iRECIST.

**Results:**

Out of 143 patients with progressive disease (PD) according to RECIST 1.1, 48 (33.6%) did not attain confirmation of progression (iCPD) as per iRECIST and six patients who were treated beyond RECIST 1.1 progression reached PD at a later point in time in iRECIST, resulting in a significant difference in TTP between iRECIST and RECIST 1.1 (618.3 ± 626.9 days vs. 538.1 ± 617.9 days, respectively (*p* < 0.05)). The number of non-responders as per RECIST 1.1 was 79, whereas it was 60 when using iRECIST. ORR was 28.5% for RECIST 1.1 and 34.1% for iRECIST, and corresponding DCR of 67.4% for RECIST 1.1 and 74.6% for iRECIST.

**Conclusion:**

iRECIST was more suitable than RECIST 1.1 for capturing atypical response patterns to ICI therapy in patients with melanoma and NSCLC, resulting in differences in the assessment of treatment response.

**Clinical relevance statement:**

Compared to RECIST 1.1, iRECIST may improve patient care and treatment decisions for patients with NSCLC or melanoma who are treated with immune checkpoint inhibitors in clinical routine.

**Key Points:**

- *RECIST 1.1 may incorrectly assess atypical treatment patterns to immune checkpoint inhibitors.*
- *iRECIST better captured atypical response patterns compared to RECIST 1.1.*
- *iRECIST was more suitable for assessing response to immune checkpoint inhibitors in non-small cell lung carcinoma and melanoma.*

## Introduction

Immune checkpoint inhibitors (ICI) targeting cytotoxic T-lymphocyte-associated protein 4 (CTLA-4), programmed cell death protein 1 (PD-1), and programmed death-ligand 1 (PD-L1) represent a major advancement in cancer therapy with significant improvements in overall survival and progression-free survival (PFS) for a broad variety of oncologic diseases such as non-small cell lung carcinoma (NSCLC) and melanoma [1–4]. New therapy response patterns uncommon in traditional cytotoxic chemotherapy have been observed more frequently in patients treated with ICI, in particular a phenomenon named pseudoprogression, which represents an initial increase in tumor burden followed by a therapy response [5–8].

With the increasing application of ICI, these atypical response patterns represent an emerging challenge for established imaging response criteria and patient management. Response evaluation criteria in solid tumors (RECIST 1.1) are the current standard widely used in clinical trials [9, 10]. Albeit not being initially conceived for application outside of clinical trials, efforts have been made to adopt RECIST 1.1, at least in part, for response evaluation in routine clinical practice and use it as a quantitative guardrail [9–11]. However, RECIST 1.1 may not assess atypical tumor response such as pseudoprogression as a treatment effect, but rather evaluate it as progressive disease (PD), resulting in potentially premature treatment discontinuation despite ongoing clinical benefit [6, 7]. As a consequence, alternative response criteria have been developed to take pseudoprogression and other atypical response patterns during ICI into consideration.

Immune-related response criteria [8] were already published in 2009 based on the World Health Organization response criteria and allowed for the continuation of response evaluation after traditional PD. This approach was further modified and adapted to the RECIST approach with its uni-dimensional measurements in 2014 with immune-related RECIST (irRECIST) [12]. However, irRECIST has been scrutinized for its ambiguity regarding the confirmation of PD, leading to different adoptions depending on the clinical trial protocols and inconsistencies across studies [13]. To address these concerns and standardize response assessment, immune RECIST (iRECIST) was published by the official RECIST working group in 2017 [14] and has since found widespread acceptance for guiding response assessment of patients under ICI therapy inside and outside of clinical trials [13].

Despite its broad acceptance, evidence is limited on whether iRECIST provides a significant advantage over RECIST 1.1 in the assessment of ICI treatment response, particularly in real-world use outside of clinical trials [15]. Although multiple studies have suggested an improved capacity of iRECIST to evaluate atypical tumor response patterns compared to RECIST 1.1 [16–19], many of them are limited to single tumor entities, relatively small sample sizes, and/or to patients treated exclusively within clinical trials.

The aim of this study was therefore to compare response assessment of iRECIST and RECIST 1.1 in patients with NSCLC and melanoma with a focus on ICI as a standard treatment in a real-world setting.

## Materials and methods

### Study cohort

After reviewing the study design, the institutional review board waived the need for informed patient consent and approved this retrospective monocenter study.

Of all patients who received ICI for cancer therapy at the University Hospital Cologne and a baseline examination between January 2015 and October 2023, those who met the following criteria were included in the study:

1. Diagnosed with histopathologically proven malignant melanoma or NSCLC and did not suffer from an additional metastatic malignancy.
2. Treated with CTLA-4 inhibitor ipilimumab, PD-1 inhibitors nivolumab and pembrolizumab, or a combination of both ipilimumab and nivolumab.
3. Did not receive other forms of chemotherapy or radiation therapy during the period observed.
4. Underwent a baseline contrast-enhanced, portal venous phase CT examination of the chest and abdomen a maximum of 12 weeks before the start of ICI therapy.
5. Underwent a minimum of two follow-up contrast-enhanced CT examinations of the chest and abdomen during the therapy period (or directly thereafter, if a new therapy had not yet been started).

### Image acquisition

The patients received contrast-enhanced examinations of the chest and abdomen either on a dual-layer detector dual-energy CT system (IQon, Philips Healthcare), a dual source dual-energy CT system (SOMATOM Force, Siemens Healthineers), a 128 slice PET/CT (Biograph mCT 128, Siemens Healthineers) or conventional CT systems (iCT 256, Philips Healthcare and Brilliance 64, Philips Healthcare, respectively). Additionally, in 24 cases, CT scans acquired in referring institutions were used at least in part for treatment response evaluation. The clinical scans were performed in cranio-caudal direction while patients remained in a supine position during inspirational breath-hold. Scans performed at our institution were reconstructed with a 2-mm slice thickness. All external scans had a slice thickness of less than or equal to 5 mm.

### Treatment response evaluation

For treatment response evaluation, the CT examinations were imported into a dedicated software for oncologic response evaluation (Mint Lesion, Mint Medical GmbH). The assessment was performed retrospectively for all baseline and follow-up examinations in accordance with the official RECIST 1.1 and iRECIST guidelines (see Table 1 for an overview of respective response criteria) [9, 14] by a board-certified radiologist with 6 years of experience in oncological imaging. Unclear evaluations were discussed with a second, highly experienced board-certified radiologist (> 15 years of experience) and the consensus decision was reported. The evaluation was conducted either until the end of the therapy interval (or directly thereafter, if a new therapy had not yet been started) or until the first follow-up examination in which PD or, in the case of iRECIST, confirmed progressive disease (iCPD), was determined.

**Table 1:** Comparison of response assessment categories between RECIST 1.1 and iRECIST

RECIST 1.1		iRECIST	
Category	Definition	Category	Definition
New lesions	Represent PD Recorded but not measured	New lesions	Represent iUPD, confirmation necessary Are not incorporated into baseline tumor burden Are categorized into new target and non-target lesions
CR	Disappearance of all target and non-target lesions AND no new lesions	iCR	Disappearance of all target and non-target lesions AND no new lesions
PR	Decrease ≥ 30% of the target sum compared to baseline AND non-unequivocal progression of non-target lesions	iPR	Decrease ≥ 30% of the target sum compared to baseline AND non-unequivocal progression of non-target lesions
SD	Neither PR nor PD	iSD	Neither iPR nor iUPD/iCPD
PD	Increase ≥ 20% of the target sum compared to nadir OR unequivocal progression of non-target lesions OR new lesions	iUPD	Increase ≥ 20% of the target sum compared to nadir OR unequivocal progression of non-target lesions OR new lesions
	Confirmation of PD is not required	iCPD	Worsening in lesion category with prior iUPD: Increase ≥ 5 mm of target sum OR any increase in non-target lesions OR further increase of new lesions (increase ≥ 5 mm of new target sum or any increase in new non-target lesions) OR new RECIST 1.1 PD in lesion category without prior iUPD
Non-CR/non-PD	No target lesions available AND neither complete disappearance nor unequivocal progression of non-target lesions	Non-iCR/Non-iUPD	No target lesions available AND neither complete disappearance nor unequivocal progression of non-target lesions

### Statistical methods

Statistical analysis was conducted using R with R Studio [20, 21] and MedCalc (MedCalc Software Ltd). Continuous variables are provided as mean with corresponding standard deviation. In accordance with the RECIST 1.1 guidelines [9], the best overall response was defined as the best treatment response recorded from the start of ICI therapy until disease progression or until the end of the therapy period, taking into account any requirement for confirmation. Overall response rate (ORR) was defined by the proportion of patients achieving either a complete (complete response (CR)/immune complete response (iCR)) or partial response (PR/iPR) as the best overall response during the course of follow-up. Correspondingly, disease control rate (DCR) was defined by the proportion of patients achieving either CR/iCR, PR/iPR, or stable disease (SD/immune stable disease (iSD)). DCR and ORR were compared between RECIST 1.1 and iRECIST evaluations using the chi-square test. Time to progression (TTP) as per RECIST 1.1 was defined as days from baseline evaluation to PD. For iRECIST, in the case of iCPD, TTP was set as the time from baseline to the immune unconfirmed progressive disease (iUPD) that was set previous to the confirmation of progression in the subsequent follow-up; in case PD was not reached as per either iRECIST or RECIST 1.1, patients were censored at the time of last available follow-up. TTP was compared between RECIST 1.1 and iRECIST using a paired Wilcoxon signed-rank test. Statistical significance was defined as *p* ≤ 0.05.

## Results

### Study cohort

A total of 252 patients (mean age at the baseline examination 62.0 ± 14.2 years), 150 men (60%, mean age 62.7 ± 14.5 years), and 102 women (mean age 60.9 ± 13.7 years), were included in the study. 177 patients (70.2%) were diagnosed with melanoma and 75 patients (29.8%) with NSCLC. Overall, 100 patients (39.7%) were treated with pembrolizumab, 82 (32.5%) with nivolumab, 8 (3.2%) with ipilimumab, 51 (20.2%) with both ipilimumab and nivolumab and 11 (4.4%) patients received two different ICI monotherapies in succession (see Table 2). Across all patients, a total of 1743 CT examinations were evaluated, with an average number of follow-up examinations during the time period of ICI therapy of 5.0 ± 3.8. The average time from baseline to first follow-up was 110 ± 47 days.

**Table 2:** Patient characteristics including sex, age, tumor subtype, and therapy regimen

Patients		252
	Men	150
	Women	102
	Age	62.0 ± 14.2 years
Tumor subtype	Melanoma	177 patients
	Non-small cell lung cancer	75 patients
Therapy regimen	Pembrolizumab	100 patients
	Nivolumab	82 patients
	Ipilimumab	8 patients
	Nivolumab + Ipilimumab	51 patients
	Pembrolizumab followed by Ipilimumab	4 patients
	Nivolumab followed by Pembrolizumab	2 patients
	Ipilimumab followed by Nivolumab	2 patients
	Ipilimumab followed by Pembrolizumab	2 patients
	Not evaluable	1 patient

### Treatment response

**Fig. 1:**
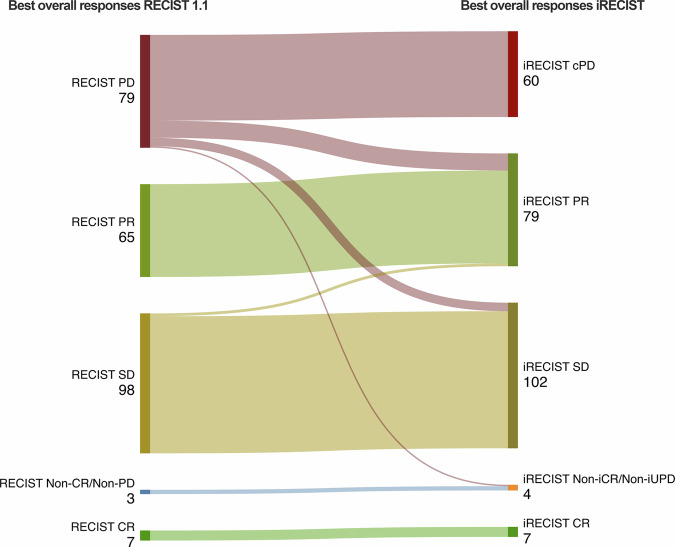
Sankey plot comparing best overall responses between RECIST 1.1 and iRECIST. 19 out of 252 patients (7.5%) who were non-responders according to RECIST (i.e., with progressive disease (PD) as their best overall response) attained a response according to iRECIST (12/19: partial remission (PR); 6/19: stable disease (SD); 1/19: non-iCR/Non-iUPD). In addition, 2 patients for which the best overall response according to RECIST 1.1 was SD received the best overall response of PR according to iRECIST

**Fig. 2:**
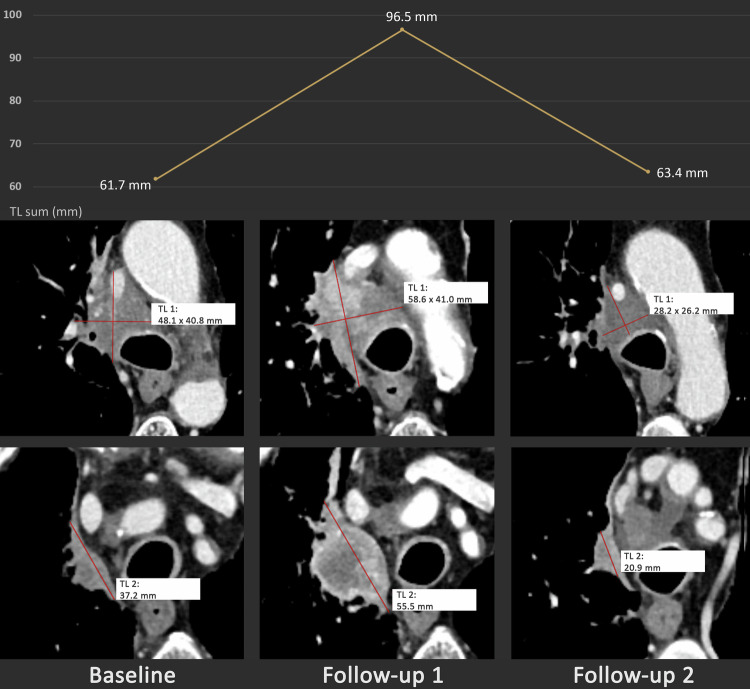
Exemplary case of discrepant response assessment between RECIST 1.1 and iRECIST. A 70-year-old male patient with metastatic non-small cell lung cancer was treated with nivolumab and showed initial progression of disease, followed by stable disease. The best overall response according to RECIST 1.1 was progressive disease, whereas it was stable disease according to iRECIST. The image depicts lymph node metastases with initial progression and subsequent tumor shrinkage. The diagram at the top shows the time course of the sum of the diameters of the target lesions (TL sum)

**Fig. 3:**
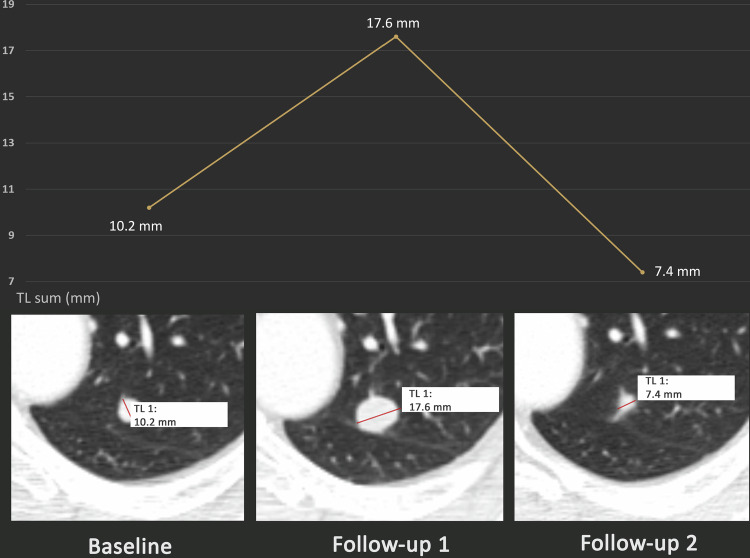
Exemplary case for a discrepant response assessment between RECIST 1.1 and iRECIST. A 58-year-old male patient with stage 4 melanoma showed initial progression of a singular lung metastasis under nivolumab monotherapy, followed by partial remission. Progression was attained at follow-up 1 according to RECIST 1.1, whereas this progression was unconfirmed according to iRECIST and subsequently transformed to partial remission. The diagram at the top shows the time course of the sum of the diameters of the target lesions

**Fig. 4:**
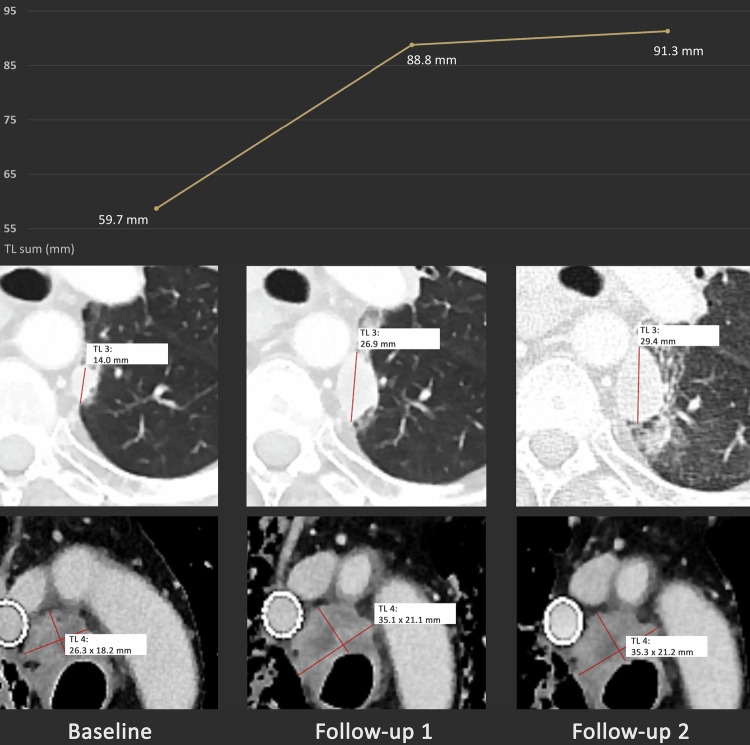
Exemplary case of a concordant response between iRECIST and RECIST 1.1. A 56-year-old man with stage 4 non-small cell lung cancer was treated with nivolumab monotherapy. The patient showed progression of disease both according to RECIST 1.1 and iRECIST. The images depict one progressive lung metastasis and one progressive mediastinal lymph node. The diagram at the top shows the time course of the sum of the diameters of the target lesions (TL sum)

The distribution of best overall responses was as follows (see Fig. 1 and Table 3): for RECIST 1.1, CR was reached in 2.8% (7/252), PR in 25.8% (65/252), SD in 38.9% (98/252), PD in 31.3% (79/252) and Non-CR/Non-PD in 1.2% (3/252) of patients. For iRECIST, iCR was reached in 2.8% (7/252), iPR in 31.3% (79/252), iSD in 40.5% (102/252), iCPD in 23.8% (60/252) and Non-iCR/Non-iUPD in 1.6% (4/252) of patients. Out of all 252 patients, 143 (56.7%) showed PD at some point in time in the period of observation according to RECIST 1.1. In this subgroup, PD was confirmed in iRECIST (iCPD) for 95 patients (66.4%), while iCPD was not attained for 48 patients (33.6%) (see also Figs. 2–4). The mean percentage change between iUPD and iCPD according to iRECIST was 24.7% ± 23.6%. For 6 patients, iCPD was reached at a later point in time compared to RECIST 1.1. With a median follow-up of 533 days, the aforementioned discrepancies led to a significant difference in mean TTP between iRECIST and RECIST 1.1 (618.3 ± 626.9 days for iRECIST vs. 538.1 ± 617.9 days for RECIST 1.1 (*p* < 0.05)). Furthermore, in iRECIST, unconfirmed progressive disease (iUPD) transitioned to iSD in the subsequent follow-ups in 15 patients and to iPR in 16 patients who have been treated beyond RECIST 1.1 progression, respectively. Of those patients, only six patients for which two or more follow-up examinations after the transition were available ultimately developed iCPD, whereas 14 patients did not attain confirmed progression at any time of their subsequent follow-up. This resulted in differences in ORR with 28.5% for RECIST 1.1 and 34.1% for iRECIST, as well as in DCR with 67.4% for RECIST 1.1 and 74.6% for iRECIST.

**Table 3:** Comparison of best overall responses between RECIST 1.1 and iRECIST

IRECIST		RECIST 1.1	
Response	Number of patients	Response	Number of patients
CR	7	CR	7
PR	79	PR	65
SD	102	SD	98
PD	60	PD	79
Non-iCR/Non-iUPD	4	Non-CR/Non-PD	3

## Discussion

Cancer treatment with novel ICI may lead to an incorrect assessment by traditional treatment response criteria such as RECIST 1.1. In this context, atypical response patterns such as pseudoprogression may be falsely evaluated as PD, potentially leading to premature discontinuation of treatment [6, 7]. For this purpose, new response criteria such as iRECIST have been developed, which require confirmation of PD in the subsequent follow-up examination according to specified criteria [14]. In this study, we investigated if and to what extent iRECIST can better account for atypical tumor response patterns of ICI therapy in patients with melanoma and NSCLC compared to RECIST 1.1.

We found that of all patients with PD according to RECIST 1.1, 31 transitioned from iUPD to PR or SD according to iRECIST in the subsequent follow-up examination, leading to an increased ORR and DCR as per iRECIST assessment. Notably, 14 of those patients with available follow-ups after that transition did not reach confirmation of progression as per iRECIST at any later point in the course of the disease. Further, a subset of patients who were treated beyond RECIST 1.1 progression reached PD at a later point in time in iRECIST compared to RECIST 1.1, representing a prolongation of TTP.

Overall, recent evidence on the potential benefit of iRECIST over RECIST 1.1 in ICI therapy response evaluation remains heterogeneous. Although numerous studies in the recent past have found a potential benefit of iRECIST over RECIST 1.1 in assessing atypical tumor response patterns, they vary significantly regarding patient cohorts, types of malignancy, and therapeutic agents. Many are limited to relatively small sample sizes, a single tumor entity and/or to patients who were treated in a clinical study, which may not be representative of real-world therapy scenarios [17, 19, 22–25].

In a recent meta-analysis, Park et al [15] included a group of 11 studies and, in contrast to our results, ultimately found no difference between RECIST 1.1 and iRECIST on response-related endpoints such as ORR and only a moderately prolonged restricted mean PFS time by 0.46 months in iRECIST. However, this could partially be caused by the heterogeneous nature of the included studies with a broad variety of tumor entities, sample sizes, and study designs. In particular, some of the included studies were entirely based on clinical trial data, further impairing comparability to the results we found.

Several retrospective studies regarding ICI treatment response evaluation comparison of patients with NSCLC in a real-world setting have been published in recent years. Tazdait et al [18] found atypical response patterns identified by iRECIST in 13% of 160 patients treated with PD-1/PD-L1-inhibitors, which, although a different measure, seems comparable to the rate of patients who did not reach iCPD in our study (19.0% of the total cohort). A comparably lower rate of 7% of a relatively small cohort of 43 patients who did not reach iCPD in iRECIST after being assessed PD in RECIST 1.1 was found by Liang et al [23]. Katz et al [26] showed a relatively low rate of 2% for pseudoprogression, correctly identified by iRECIST, in 160 patients treated with PD-1/PD-L1-inhibitors nivolumab or pembrolizumab. These three studies however did not compare survival times, ORR, or DCR of the total collective between RECIST 1.1 and iRECIST.

With regard to melanoma, in line with our results, Ahmed et al found a discordance of treatment response in iRECIST compared to RECIST 1.1 in a cohort of 221 patients treated with pembrolizumab with a rate of discordance in the best overall response of 4.5% as well as a rate of pseudoprogression of 6.3% among the entire cohort [16]. Although we used different measures, this seems lower than the results in our study, where 33.6% of patients with PD according to RECIST 1.1 did not reach iCPD in iRECIST. However, Ahmed et al only included patients who were treated in the context of a clinical trial, which may not be representative of the real-world therapy scenario.

To our knowledge, our study represents the first large-scale study comparing ICI treatment response in RECIST 1.1 and iRECIST in a large cohort of patients with melanoma and NSCLC with a focus on the real-world setting. We could show that a significant number of cases with PD in RECIST 1.1 remained unconfirmed in iRECIST, often leading to disease stabilization or iPR with continued further treatment. Therefore, our results underline the better suitability of iRECIST for the evaluation of atypical response patterns in patients treated with ICI within the clinical routine. This has important practical implications since the assessment of response during ICI by iRECIST may prevent premature discontinuation of ICI, thereby enabling prolonged treatment and potentially improved outcomes. Further, the concept of confirmation of PD in iRECIST leads to discrepancies in clinical outcome parameters such as mean TTP as well as ORR and DCR compared to RECIST 1.1, which has to be kept in mind when interpreting results from studies—both prospective and retrospective—relying on those criteria. On the contrary, these potential benefits of iRECIST in the small subset of patients with atypical response patterns have to be weighed against the additional financial cost of additional imaging and medication and the expenditure of time on potentially ineffective therapy.

Apart from the inherent limitations of a retrospective monocenter study design, there are other potential limitations to our study, which need to be addressed. First, the comparison of RECIST 1.1 and iRECIST is limited by the fact that for 17 patients, after RECIST 1.1 PD, therapy was discontinued, and therefore it remains elusive if the progression would have been confirmed according to iRECIST in the scenario of treatment beyond progression. On the contrary, this shows that discontinuation or change of therapy after PD according to RECIST 1.1 or in the corresponding radiological report occurred in a relevant number of cases, which, in the light of the results we found, further underlines that a continuation with short term follow-up might be the better approach for the clinical management of those patients if they show continued clinical benefit. Conversely, an assessment analogous to iRECIST in clinical routine with the necessity to confirm disease progression bears the risk of delayed diagnosis of true progressions. Assessing the latter was out of the scope of our study and should be encompassed in subsequent evaluations. Further, we did not assess response patterns separately for melanoma and NSCLC due to the small sample size in these subsets, potentially masking tumor-specific differences in treatment response. However, it has to be taken into consideration that tumors of the same entity may also significantly differ interindividually in terms of their biological composition. While this was out of the scope of this study, we would encourage future studies highlighting this and other entity-specific questions. Third, retrospective response evaluation in RECIST 1.1 and iRECIST, as shown in this study, may be not entirely comparable to prospective response evaluation parallel to the treatment course in a real-world setting. Therefore, we encourage further prospective studies on this topic.

## Conclusions

We found significant discrepancies between RECIST 1.1 and iRECIST in radiological response evaluation of ICI therapy in clinical routine. iRECIST more accurately captured atypical response patterns, leading to a longer measured TTP and higher ORR as well as DCR. Subsequent studies should prospectively evaluate whether capturing such responses outweighs the potential risk of a delayed diagnosis due to the necessity of confirming disease progressions.

## Abbreviations

CR: Complete response
CTLA-4: Cytotoxic T-lymphocyte-associated protein 4
DCR: Disease control rate
ICI: Immune checkpoint inhibitors
iCPD: Immune confirmed progressive disease
iCR: Immune complete response
iPR: Immune partial response
iRECIST: Immune response evaluation criteria in solid tumors
irRECIST 1.1: Immune-related response evaluation criteria in solid tumors 1.1
iSD: Immune stable disease
iUPD: Immune unconfirmed progressive disease
NSCLC: Non-small cell lung cancer
ORR: Overall response rate
PD: Progressive disease
PD-1: Programmed cell death protein 1
PD-L1: Programmed death-ligand 1
PFS: Progression-free survival
PR: Partial response
RECIST 1.1: Response evaluation criteria in solid tumors 1.1
SD: Stable disease
TTP: Time to progression
